# Transcriptional changes in LMH cells induced by *Eimeria tenella* rhoptry kinase family protein 17

**DOI:** 10.3389/fvets.2022.956040

**Published:** 2022-08-09

**Authors:** Yi-Jing Meng, Bing-Jin Mu, Xiao-Xin Liu, Lin-Mei Yu, Wen-Bin Zheng, Shi-Chen Xie, Wen-Wei Gao, Xing-Quan Zhu, Qing Liu

**Affiliations:** ^1^College of Veterinary Medicine, Shanxi Agricultural University, Jinzhong, China; ^2^Key Laboratory of Veterinary Public Health of Higher Education of Yunnan Province, College of Veterinary Medicine, Yunnan Agricultural University, Kunming, China

**Keywords:** *Eimeria tenella*, rhoptry kinase family protein 17, LMH cells, overexpression, transcriptome

## Abstract

Though a number of *Eimeria tenella* rhoptry kinase family proteins have been identified, little is known about their molecular functions. In the present study, the gene fragment encoding the matured peptide of *E. tenella* rhoptry kinase family protein 17 (EtROP17) was used to construct a recombinant vector, followed by transfection into leghorn male hepatoma (LMH) cells. Then, the transcriptional changes in the transfected cells were determined by RNA-seq. The expression of EtROP17 in LMH cells was validated by both Western blot and indirect immunofluorescence analysis. Our analysis showed that EtROP17 altered the expression of 309 genes (114 downregulated genes and 195 upregulated genes) in LMH cells. The quantitative real-time polymerase chain reaction (qRT-PCR) results of the selected differentially expressed genes (DEGs) were consistent with the RNA-seq data. Kyoto Encyclopedia of Genes and Genomes (KEGG) analysis showed that DEGs were significantly enriched in nine pathways, such as toll-like receptor signaling pathway, ECM-receptor interaction, intestinal immune network for IgA production and focal adhesion. These findings reveal several potential roles of EtROP17, which contribute to understanding the molecular mechanisms underlying the host-parasite interplay.

## Introduction

Avian coccidiosis is an economically significant disease of the poultry industry caused by intracellular intestinal parasites of the genus *Eimeria*, with a worldwide distribution (1, 2). Seven *Eimeria* species, namely *Eimeria maxima, Eimeria acervulina, Eimeria necatrix, Eimeria brunetti, Eimeria mitis, Eimeria tenella* and *Eimeria praecox*, are recognized to infect chickens (3). *E. tenella* is one of the most pathogenic species, causing caecal coccidiosis of chickens (4, 5).

Twenty-eight rhoptry kinase family proteins (ROPs) have been predicted in *E. tenella* through genomic analysis, such as *E. tenella* ROP 17 (EtROP17), EtROP21 and EtROP30 (6). Subsequently, the expression patterns of these genes during the *E. tenella* life-cycle have been studied by quantitative real-time polymerase chain reaction (qRT-PCR) (7). Meanwhile, EtROP30 was reported to localize to the nucleus and possibly play an important role during parasite reinvasion and development (8). Additionally, a previous study showed that Et-ROPK-Eten5-A may be a potential candidate for the development of new vaccines against *E. tenella* (9). To date, however, the roles of the majority of EtROPs remain unknown.

*Toxoplasma gondii* ROP17 (TgROP17) was reported to be an important effector molecule, which is associated with maintaining *T. gondii* proliferation in host cells through regulating the Bcl-2-Beclin 1 pathway (10). Moreover, TgROP17 contributes to *T. gondii* dissemination (11). Additionally, transcriptomic analysis showed that TgROP17 plays a pivotal role in the survival of *T. gondii* within host cells by inhibiting the innate immune response (12).

**Figure 1:**
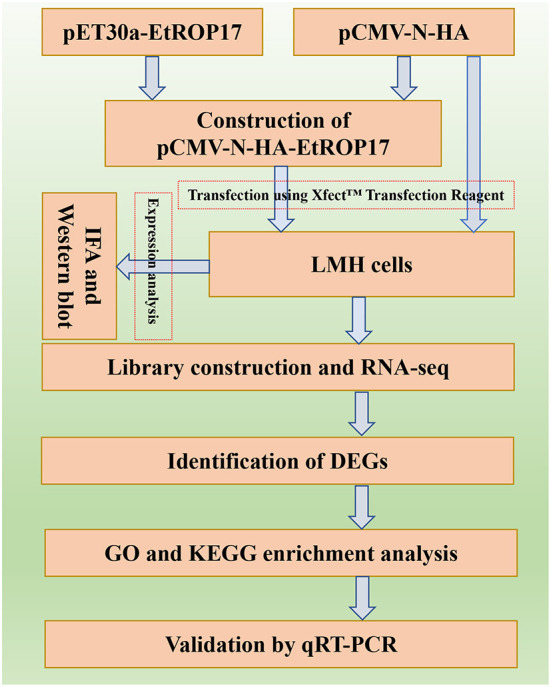
Flow chart of the performed research work.

The aim of the present study was to determine the biological function of EtROP17, which shares homology with TgROP17. To achieve this, the fragment of the ROP17 gene of the *E. tenella* SD-01 strain was used to construct the recombinant vector. Then, the role of EtROP17 was determined by transcriptomic analysis of leghorn male hepatoma (LMH) cells transfected with the recombinant vector. The workflow of the research is illustrated in Figure 1.

## Materials and methods

### Cell line

An epithelial cell line, LMH, was used for transfection, which was often used in studies of avian pathogens (13, 14). LMH cells were grown in Dulbecco's modified eagle medium (DMEM) containing 10% (vol/vol) fetal bovine serum (FBS), 100 U/mL penicillin, and 100 μg/mL streptomycin. The cell culture was maintained at 37°C in an incubator with 5% CO_2_, and cells were passaged every 2 days (15).

### Construction of the recombinant vector

**Figure 2:**
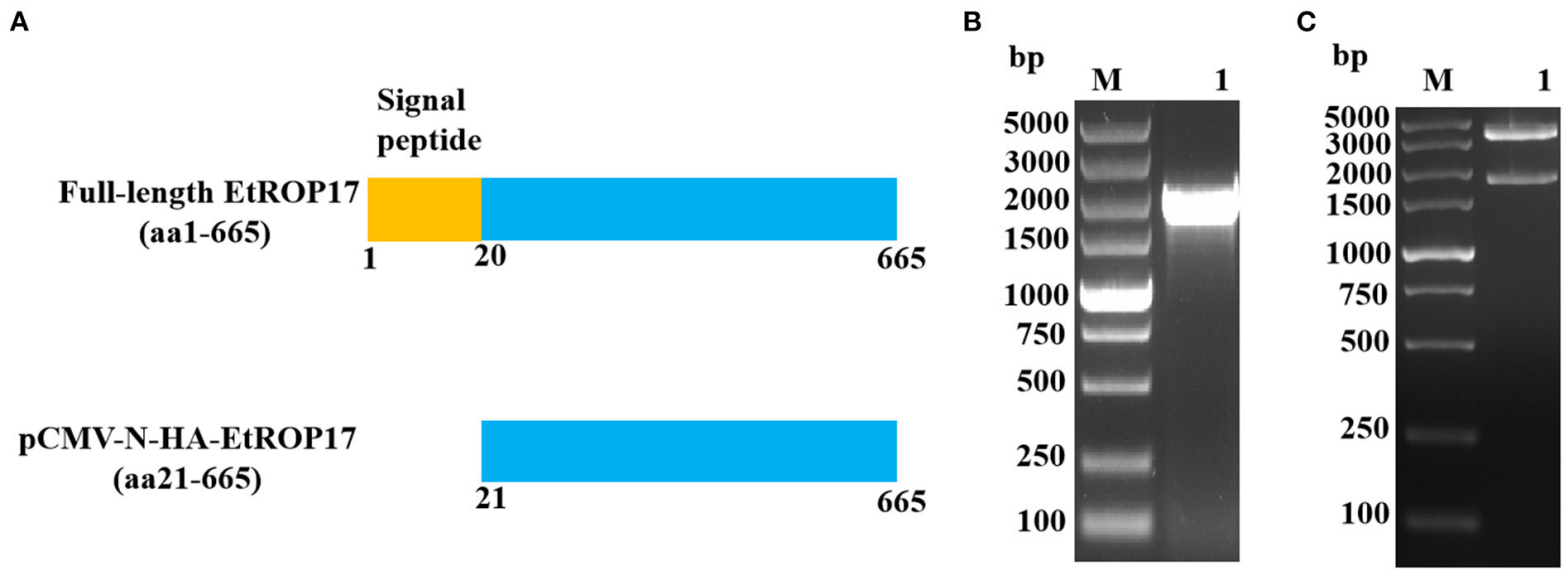
**(A)** Schematic illustration of full-length EtROP17 and the region of EtROP17 used in the present study. **(B)** The gel electrophoresis of the PCR products. Lane M, DL5000 DNA Marker; lane 1, the gene fragment of EtROP17 amplified from cDNA of *E. tenella* SD-01 strain. **(C)** Digestion of the resultant vector with BamHI and EcoRI restriction enzymes.

The pCMV-N-HA vector and the previously constructed pET30a-EtROP17 were double digested with BamHI and EcoRI (16), and the nucleotide sequence encoding the matured peptide of EtROP17 (Figure 2A) was ligated into the linearized vector pCMV-N-HA using the T4 DNA Ligase (Takara, Dalian, China). Following transformation into *Escherichia coli* DH5α competent cells (Transgen Biotech, China), single bacterial colony was randomly selected for PCR analysis. The positive colony was cultured in Luria-Bertani (LB) medium, and the recombinant vector (designated pCMV-N-HA-EtROP17) was extracted using the PhasePrep EndoFree Maxi Kit (Aidlab, Beijing, China) according to the manufacturer's specifications. The extracted vector was verified by double restriction enzyme digestion and sequencing (Sangon Biotech, Shanghai, China). The quality and quantity of yielded vector were determined by a spectrophotometer (NanoDrop One/One^c^, Thermo Scientific).

### Transfection of LMH cells

The Xfect™ Transfection Reagent (Takara, Dalian, China) was used for transfection of LMH cells (~ 80% confluent). For immunofluorescence analysis, 1 day prior to the transfection, the LMH cells were seeded in 24-well plate. Then, 0.75 μg PCMV-N-HA-EtROP17 and PCMV-N-HA were diluted separately with Xfect reaction buffer to a final volume of 25 μL. Afterwards, 0.225 μL Xfect polymer was added and incubated for 10 min at room temperature to allow nanoparticle complexes to form. The mixture was then added to the cell culture medium and incubated for 4 h. Subsequently, the medium was replaced with 500 μL fresh DMEM plus 10% FBS.

For Western blot analysis and RNA-seq, the LMH cells were seeded in T-25cm^2^ cell culture flasks, and 12.5 μg PCMV-N-HA-EtROP17 and PCMV-N-HA were used separately for transfection. Meanwhile, each plasmid was diluted with Xfect reaction buffer to a final volume of 250 μL, and 3.75 μL Xfect polymer was used.

### Indirect immunofluorescence assay

The cells were fixed at 48 h post-transfection with 2% paraformaldehyde for 10 min, followed by incubation with 0.1% Triton X-100 for 10 min. After blocking with 5% bovine serum albumin, mouse anti-HA tag antibody (Invitrogen, CA, USA) was added to each well and incubated for 1 h. Following washing for three times with PBS, each well was incubated with FITC-conjugated goat anti-mouse IgG (Abcam, UK) at 37 °C for 1 h. The cells were observed under a Nikon fluorescence microscope (Nikon, Japan).

### Western blot

Forty-eight hours post transfection, the cells were lysed by treatment with RIPA lysis buffer (Beyotime, China). Protein extracts were electrophoretically separated under denaturing conditions with 10% Expressplus™ PAGE Gels (GenScript, China), and then blotted onto a polyvinylidene fluoride (PVDF) membrane (Millipore, USA). After blocking, the membrane was incubated with mouse anti-HA tag antibody for 2 h. After washing thrice with TBST buffer (20 mM Tris-HCl, 150 mM NaCl, 0.05% Tween 20), the membrane was incubated with HRP-conjugated goat anti-mouse IgG antibody for 1 h. The band was visualized with an enhanced chemiluminescent (ECL) reagent (Thermo Scientific, USA).

### Transcriptome sequencing and read alignment

We performed transcriptomic analysis of LMH cells transfected with PCMV-N-HA-EtROP17 or PCMV-N-HA, and the RNA-seq service was provided by Novogene Corporation (Beijing, China). Three biological replicates were included for each condition, and the TRIzol reagent (Invitrogen, CA, USA) was used for extraction of total RNA. The Agilent 2100 Bioanalyzer (Agilent Technologies, CA, USA) was used to evaluate the quality of the extracted RNA. mRNA was purified with poly-T oligo-attached magnetic beads and segmented into small fragments using divalent cations under elevated temperature, followed by cDNA synthesis. After adenylation, NEBNext Adaptor with hairpin loop structure was ligated to the cDNA fragments. Then, these fragments were subjected to purification and PCR amplification. Following purification of PCR products, quality assessment and cluster generation, the libraries were sequenced using an Illumina Novaseq platform. After removal of low quality reads and reads containing adaptor or poly-N, clean data with high quality were aligned to the chicken (*Gallus gallus*) genome (https://www.ncbi.nlm.nih.gov/genome/?term=Gallus+gallus) using Hisat2 (v2.0.5) (17).

### Bioinformatics analysis

Differential expression analysis of two groups was carried out by using the DESeq2 R package (v1.20.0) (18). Differential gene screening was performed under the condition of both *P* value <0.05 and |log_2_ fold change (FC)|>1.0. Gene Ontology (GO) annotation and Kyoto Encyclopedia of Genes and Genomes (KEGG) pathway enrichment analysis were performed by using the clusterProfiler R package (v3.8.1) (19). A *P* value <0.05 was set as the cut-off threshold to identify the significantly enriched GO terms or pathways.

### Validation of RNA-seq data by qRT-PCR

RNA-seq results were verified by qRT-PCR, which was performed on a CFX Connect Real-Time PCR Detection System (Bio-Rad, CA, USA). Eight DEGs were selected for validation. For normalizing the expression of gene, GAPDH was used as the endogenous reference gene, and the control was used as a reference sample, which was set to one. The qRT-PCR cycling conditions included 95°C for 30 s, followed by 40 cycles of 95°C for 15 s, and 60°C for 30 s. The temperatures of the melting curve analysis ranged from 65°C to 95°C. The qRT-PCR reactions were carried out in triplicates. All the primers are shown in Table 1. Normalized FC (2^−Δ*ΔCt*^) in expression was determined based on ΔCt (Ct_target_ _gene_ – Ct_GAPDH_) and ΔΔCt (ΔCt_experimental group_ – ΔCt_controlgroup_) (20), and then the log_2_ FC value was calculated.

**Table 1:** Sequences of the primers used for the qRT-PCR assay.

**Gene ID**	**Gene name**	**Forward primer (5^′^-3^′^)**	**Reverse primer (5^′^-3^′^)**
374193	GAPDH	CTGGGGCTCATCTGAAGGGT	GGACGCTGGGATGATGTTCT
395908	OASL	GGTGCTCTTCATCAACTGCTTCTCCA	TCGTAAGCAGGCAGGATGTC
403120	IFI6	TCCTTCTGGAGGGACTACTGCTA	TGGACCGCTGCTTCTTTCTATT
418982	MMP1	TTGATGAGGAGGAAACCTGGAC	GGTCTGTGTAGGCATAGTTTGGATA
428310	HSPB9	ACGCAGAACACGGACGAGAA	TTTGCTGACAGCTCCATCCTT
428650	RSAD2	TGCCGAGATTATGCTGTTGCT	CAATGATTAGGCACTGGAACACC
395313	MX1	CTCTGCCAAAGTTGAAGAAATCG	CCTCAAATGTCCAGTAGCTGATAAAG
417964	EMP1	GTTTGGATGGTGGGTAGGAGTT	TGATGGCTCCAGTGATGTAGAAAC
428431	GPNMB	GAAGACCTTTCCCTCATTATCCT	CCGAGAGTGATATTTGCAGTGTT

## Results

### Confirmation of the resultant vector

As shown in Figure 2B, the gene fragment of EtROP17 was obtained by PCR amplification. Digestion of the resultant vector with BamHI and EcoRI restriction enzymes was carried out to confirm the presence of the gene fragment of EtROP17. Two bands were observed, indicating that the recombinant vector was successfully constructed (Figure 2C).

### Expression analysis of EtROP17 in LMH cells

**Figure 3:**
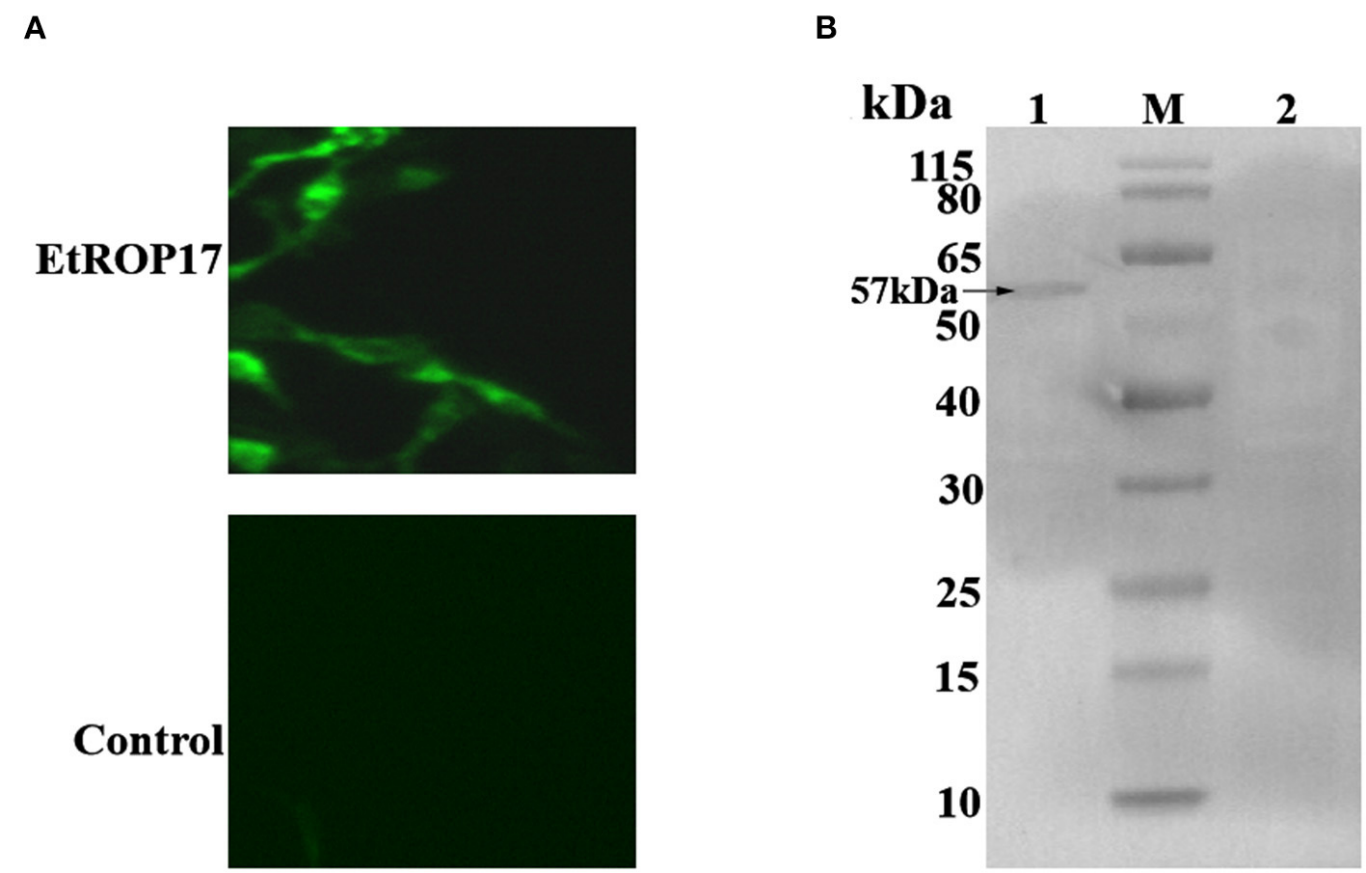
**(A)** Immunofluorescent analysis of PCMV-N-HA-EtROP17-transfected cells and PCMV-N-HA-transfected control cells. **(B)** Western blot analysis of PCMV-N-HA-EtROP17-transfected cells and PCMV-N-HA-transfected control cells. Lane 1, total protein extracted from PCMV-N-HA-EtROP17-transfected LMH cells; lane 2, total protein extracted from PCMV-N-HA-transfected control cells; the arrow indicates the position of a 57-kDa EtROP17 band.

Successful transfection was determined by means of the indirect immunofluorescence assay. As shown in Figure 3A, mouse anti-HA tag antibody clearly labeled the LMH cells transfected with pCMV-N-HA-EtROP17. Meanwhile, no fluorescent signal was observed in null plasmid transfected cells. Western blot analysis showed that LMH cells transfected with pCMV-N-HA-EtROP17 expressed a protein at a size of approximately 57 kDa (Figure 3B, lane 1), which was not observed in LMH cells transfected with the empty control vector (Figure 3B, lane 2).

### Analysis of differential gene expression

**Figure 4:**
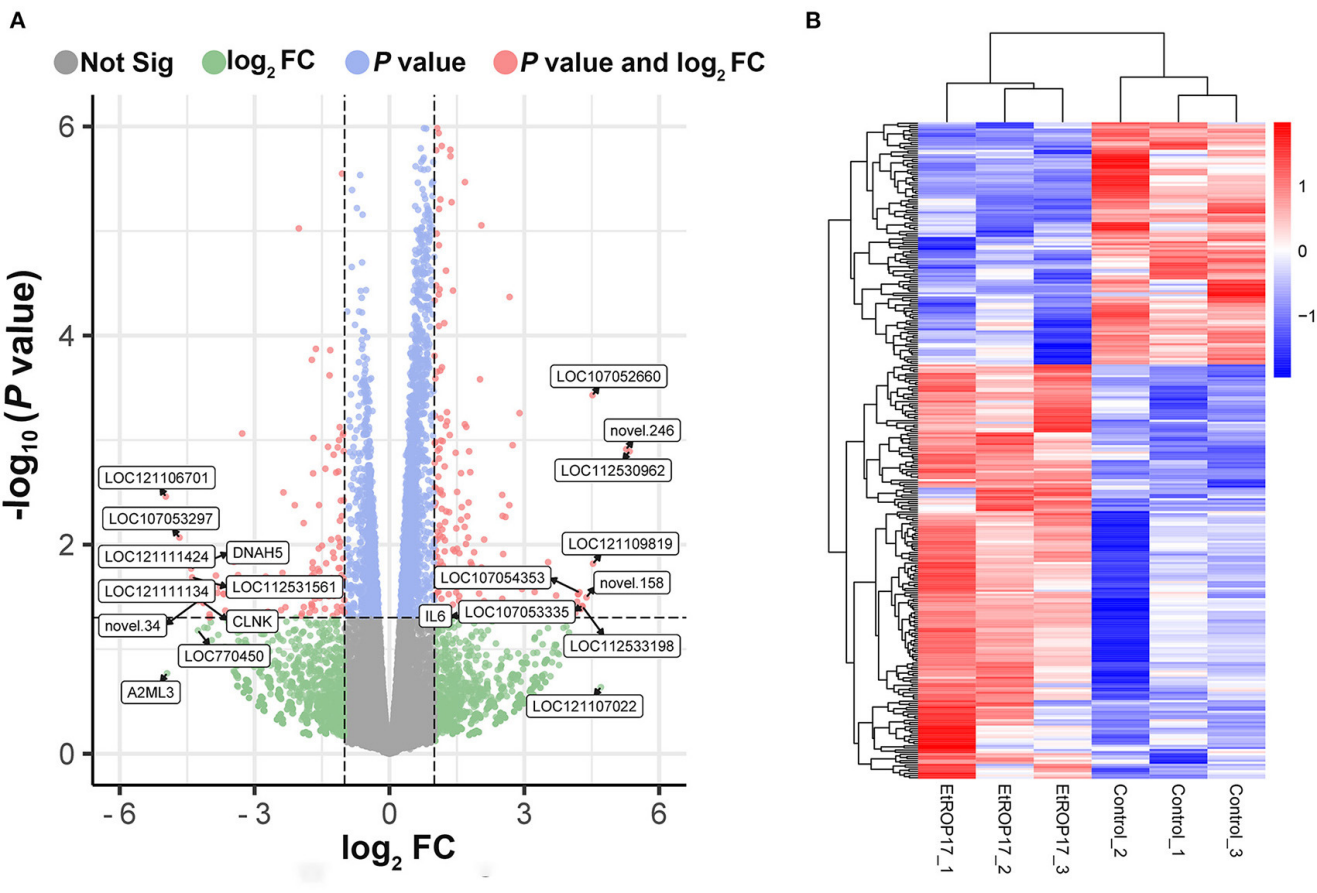
Effect of EtROP17 on gene transcription in LMH cells. **(A)** Volcano plot showing the DEGs in EtROP17-expressing LMH cells compared to the control group. **(B)** Heatmap of 309 DEGs. Rows: DEGs; columns: samples.

RNA integrity of all samples was evaluated, with RNA integrity number (RIN) values ranging from 8.9 to 10 (Supplementary Table 1). Compared with the control group, 309 genes were found to be differentially expressed in LMH cells transfected with EtROP17 (Figure 4). Of which, 195 DEGs were upregulated, and 114 DEGs were downregulated. Details of the DEGs are provided in Supplementary Table 2.

### GO enrichment

**Figure 5:**
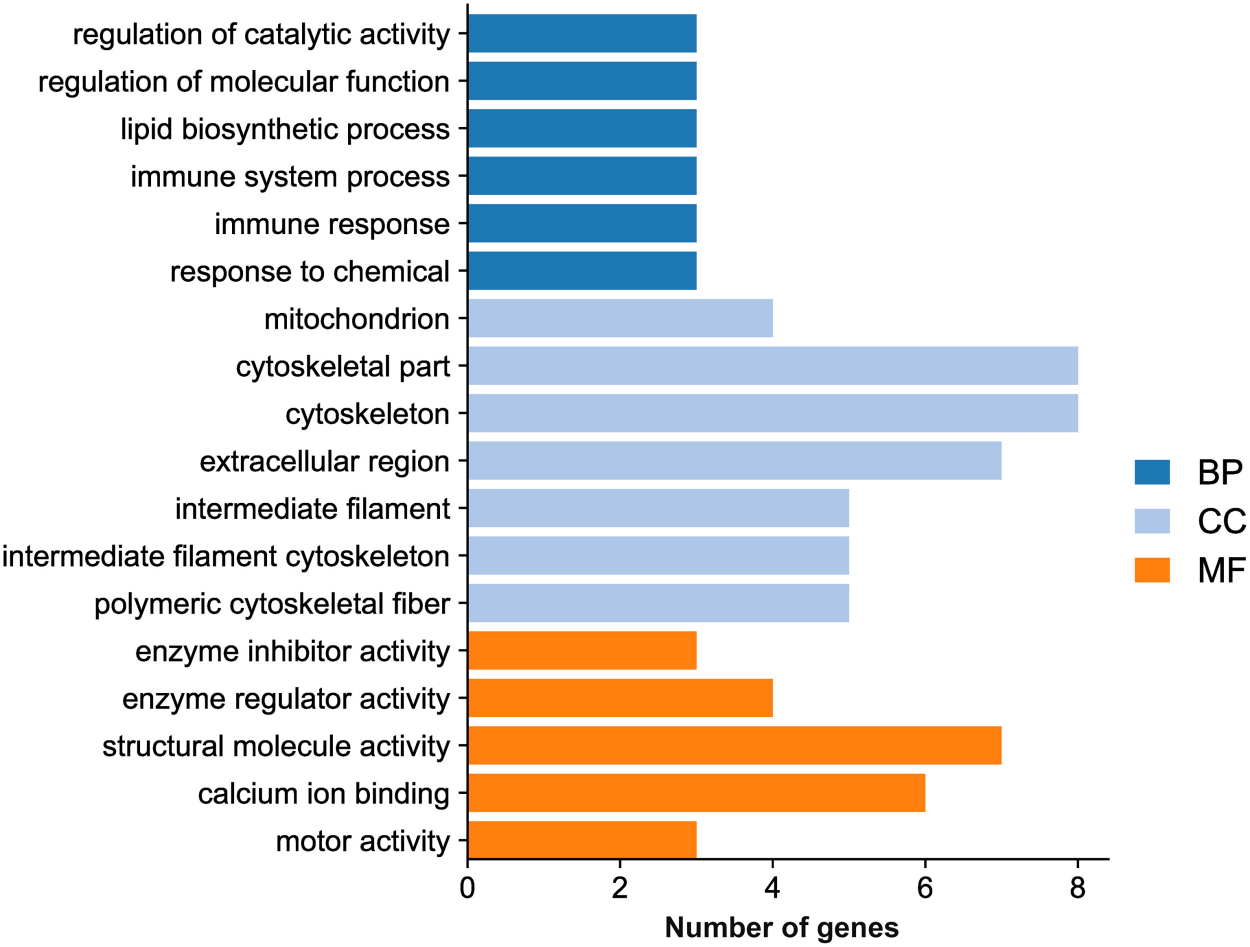
Histogram of the significantly enriched GO terms.

GO analysis showed that the DEGs were categorized into 323 GO terms. Eighteen significantly enriched terms were identified based on a *P* value <0.05 as a cutoff, including six GO biological process terms (regulation of catalytic activity, regulation of molecular function, lipid biosynthetic process, immune system process, immune response, and response to chemical), seven GO cellular component terms (mitochondrion, cytoskeletal part, cytoskeleton, extracellular region, intermediate filament, intermediate filament cytoskeleton, and polymeric cytoskeletal fiber), and five GO molecular function terms (enzyme inhibitor activity, enzyme regulator activity, structural molecule activity, calcium ion binding, and motor activity) (Figure 5; Supplementary Table 3).

### KEGG analysis

**Figure 6:**
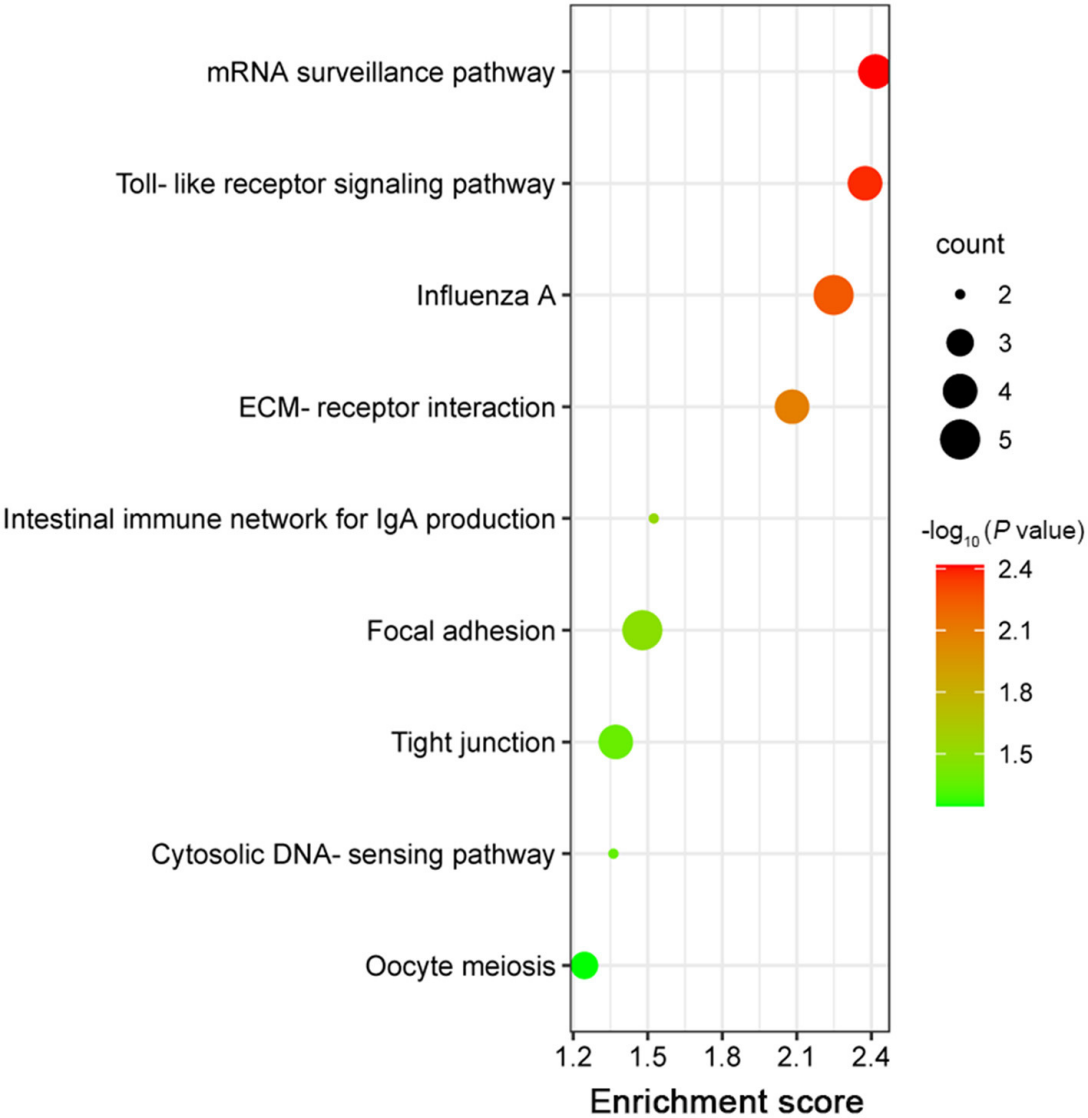
The nine significantly enriched KEGG pathways for the DEGs.

KEGG analysis of DEGs was conducted to determine the affected pathways. As shown in Figure 6, nine significantly enriched pathways were found on the basis of a *P* value <0.05 as a cutoff, namely mRNA surveillance pathway, toll-like receptor signaling pathway, influenza A, ECM-receptor interaction, intestinal immune network for IgA production, focal adhesion, tight junction, cytosolic DNA-sensing pathway, and ribosome.

### Validation of DEGs by qRT-PCR

**Figure 7:**
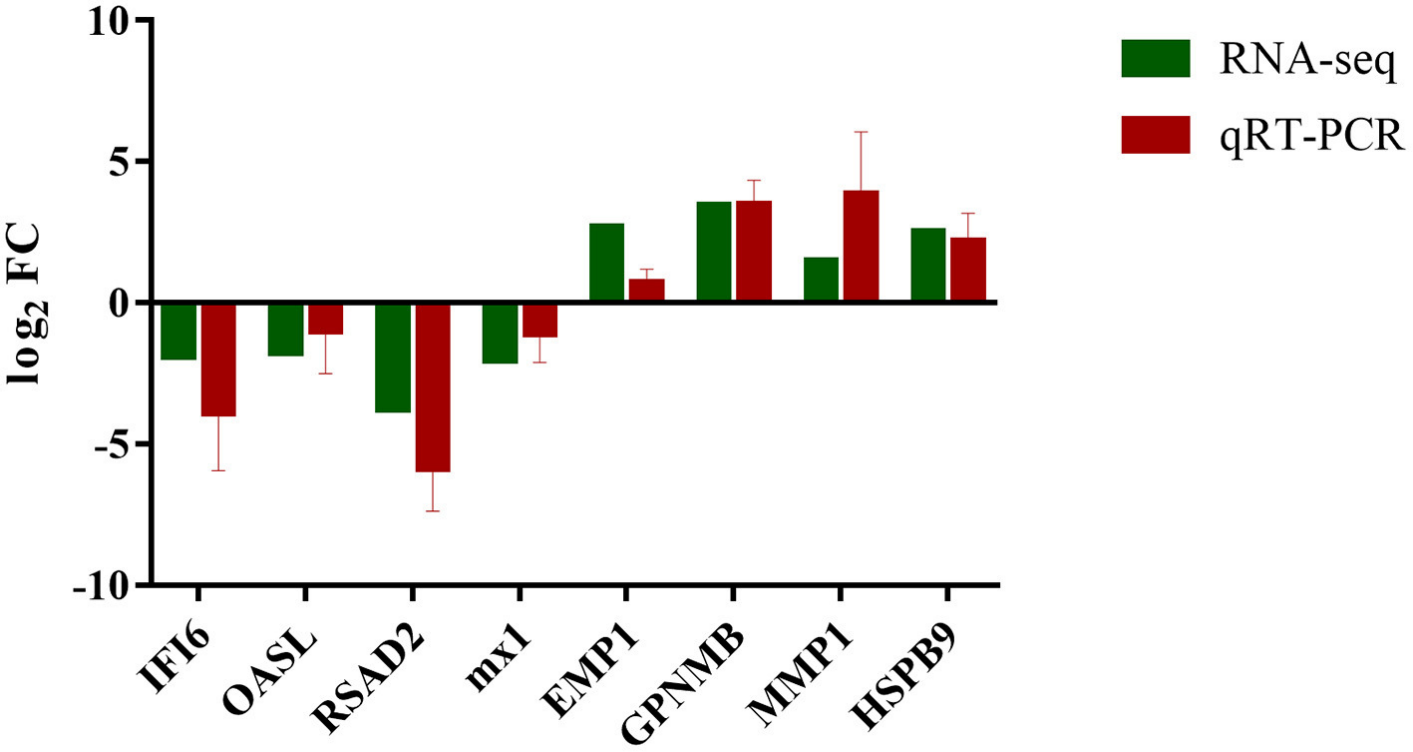
Expression verification of eight genes in RNA-seq using qRT-PCR. Green color refers to the result of RNA-seq; red color refers to the result of qRT-PCR.

The transcript expression patterns derived from RNA-Seq were validated by examining the level of expression of selected DEGs from each group using qRT-PCR. An agreement between the results obtained by RNA-seq and qRT-PCR was found (Figure 7; Supplementary Table 4).

## Discussion

*E. tenella* was reported to manipulate many signaling pathways of host cells (21, 22). To date, however, the roles of *E. tenella* proteins in host-parasite interactions remain incompletely understood. In a previous study, we confirmed the expression of EtROP17 in the merozoite stage of *E. tenella* (16). The present study aimed to provide a comprehensive picture of transcriptomic changes in host cells in response to EtROP17 based on an overexpression mode in conjunction with RNA-seq, by which the functions of several genes of *T. gondii* have been characterized (12, 23, 24). The expression of EtROP17 in LMH cells was confirmed by immunofluorescence measurements and Western blot analysis. All RNA samples showed high RIN values, and the transcriptomic data were validated by qRT-PCR. Clustering analysis showed that distinct changes in the expression genes were observed between the two groups.

A previous study revealed the roles of TgROP17 in regulation of the host immune response (12). Though the number of DEGs in LMH cells in response to EtROP17 was lower than that in HEK293T cells in response to TgROP17 (12), several DEGs identified in the present study were also involved in immune responses, such as CCL4, IL6 and IL12B. Meanwhile, GO analysis uncovered that several significantly enriched GO terms were related to immune responses, such as cytokine activity, cytokine receptor binding, immune system process, and immune response.

The host innate and adaptive immune responses, relying on a complex network of various immune cells and their signals, are involved in fighting against *E. tenella* infection (25, 26). A previous study showed that TgROP17 could negatively regulate toll-like receptor signaling pathway, which is one of the innate host defense mechanisms against pathogens (12, 27). Also, our analysis showed that toll-like receptor signaling pathway in EtROP17-expressing LMH cells was downregulated, and the related genes were CCL4 and IL12B. This indicated that *E. tenella* may negatively regulate toll-like receptor signaling pathway to inhibit innate immune responses by using EtROP17. Additionally, three downregulated DEGs (RSAD2, MX1 and IL12B) were enriched in influenza A, which is also associated with innate immune response (28).

Using RNA-seq, a previous study reported that *E. tenella* could affect intestinal immune network for IgA production in chicken cecal epithelia (29). Meanwhile, *E. maxima* was reported to be able to alter the expression of immune network for IgA production-associated genes (30). Given that IgA production may not play an important role in combating coccidian infection (29), EtROP17 is possibly involved in defense against bacterial and viral infections through affecting intestinal immune network for IgA production (31).

High-throughput sequencing results revealed that focal adhesion and ECM-receptor interaction were significantly enriched. ECM is a complex mixture composed of structural proteins, and attachment to ECM is essential for survival of epithelial cells (32, 33). Focal adhesion is involved in mediating the regulatory effects of a cell in response to ECM adhesion (34). This indicated that EtROP17 may play a crucial role in parasite replication within chicken intestinal epithelial cells. Moreover, the cell to ECM interaction can form physical barriers to prevent invasion of pathogens (35). This indicated that, besides affecting intestinal immune network for IgA production, EtROP17 may contribute to defense against microbial infections through affecting ECM-receptor interaction.

## Conclusion

The present research revealed gene expression in LMH cells in response to EtROP17 expression. A total of 114 downregulated genes and 195 upregulated genes were identified in EtROP17-expressing LMH cells, which were significantly enriched in nine signaling pathways. These data reveal several potential roles of EtROP17 and contribute to better understanding of the molecular mechanisms underlying the host-parasite interactions.

## Data availability statement

The datasets presented in this study can be found in online repositories. The names of the repository/repositories and accession number(s) can be found below: https://www.ncbi.nlm.nih.gov/bioproject/PRJNA831776.

## Author contributions

QL and X-QZ conceived and designed the experiments. Y-JM performed the experiments, analyzed the data, and wrote the paper. B-JM, X-XL, L-MY, S-CX, and W-WG participated in the implementation of the study. W-BZ, QL, and X-QZ critically revised the manuscript. All authors contributed to the article and approved the submitted version.

## Funding

Project support was provided by the National Natural Science Foundation of China (Grant No. 31902298), the Fund for Shanxi 1331 Project (Grant No. 20211331-13), the Research Fund for Introduced High-level Leading Talents of Shanxi Province, the Special Research Fund of Shanxi Agricultural University for High-level Talents (Grant No. 2021XG001) and Yunnan Expert Workstation (Grant No. 202005AF150041).

## Conflict of interest

The authors declare that the research was conducted in the absence of any commercial or financial relationship that could be construed as a potential conflict of interest.

## Publisher's note

All claims expressed in this article are solely those of the authors and do not necessarily represent those of their affiliated organizations, or those of the publisher, the editors and the reviewers. Any product that may be evaluated in this article, or claim that may be made by its manufacturer, is not guaranteed or endorsed by the publisher.
